# Penile length and circumference dimensions: A large study in young Italian men

**DOI:** 10.1111/and.14053

**Published:** 2021-03-21

**Authors:** Marina Di Mauro, Camilla Tonioni, Andrea Cocci, Luis A. Kluth, Giorgio Ivan Russo, Juan Gomez Rivas, Giovanni Cacciamani, Gianmartin Cito, Girolamo Morelli, Gaia Polloni, Fabrizio di Maida, Daniel Giunti, the Trauma, Reconstructive Urology, Men’s Health Working Parties of the European Association of Urology (EAU) Young Academic Urologists (YAU)

**Affiliations:** ^1^ Urology Section Department of Surgery University of Catania Catania Italy; ^2^ Centro Integrato di Sessuologia Il Ponte Florence Italy; ^3^ Department of Urology University of Florence Florence Italy; ^4^ Department of Urology University Hospital Frankfurt Goethe University Frankfurt am Main Germany; ^5^ Department of Urology La Paz University Hospital Madrid Spain; ^6^ USC Institute of Urology and Catherine and Joseph Aresty Department of Urology University of Southern California Los Angeles CA USA; ^7^ Urology Department University of Pisa Pisa Italy; ^8^ Private psychologist Como Italy

**Keywords:** dimensions of penis, erectile dysfunction, penile circumference, penile length, penile size

## Abstract

The aim of the present study was to evaluate the size of the penis in flaccidity and in erection of Italian men. A total of 4,685 men living in Italy and who have been visited at the Italian urology operating units were involved in the study between January 2019 and January 2020. Each patient was given details on how to measure their penis (erect length and circumference) in flaccidity and in erection, from the lower base to the distal penile tip. Mean (standard deviation [*SD*]) flaccid penis length was 9.47 (2.69), mean (*SD*) flaccid penis circumference was 9.59 (3.08), mean (*SD*) erect penis length was 16.78 (2.55) and mean (*SD*) erect penis circumference was 12.03 (3.82). At the linear regression analysis, height was associated with flaccid penis length (*β* = 0.04; *p*‐value = .01), and erect penis length was (*β* = 0.05; *p*‐value < .01) and erect penis circumference was (*β* = 0.06; *p*‐value < .01). Height is proportional to the length of the penis in flaccidity and in erection, and to the circumference in erection. The increase in BMI leads to a reduction in the length of the erect penis, as well as weight gain reduces the length of the flaccid penis.

## 1 INTRODUCTION

In the male population, penile size is gaining importance together with the length, and the number of patients needing andrological investigation is increasing (Mondaini et al., 2002; Singal & Jain, 2016). Penile size has traditionally been associated with increased sexual power, virility and vigour in men (Shalaby et al., 2015; Veale, Miles, Read, Troglia, Carmona, et al., 2015) and is closely related to man's self‐esteem. However, penis size is taboo in our society and in most cases the measurement is taken subjectively for comparison with colleagues or friends. So, identifying normality is a challenge and depends on the culture, race and form of measurement applied. Interestingly, Park et al. have previously demonstrated that fourth digit ratio, flaccid penile length and age of circumcision were significant predictive factors for erectile penile length (Park et al., 2016). Furthermore, a survey of over 52.000 subjects revealed that 85% of women were satisfied with their partner's penis size. However, only 55% of men were satisfied with the size of one's penis (Lever et al., 2006). It, therefore, appears that men tend to underestimate their dimensions and that they are more interested in their size than women. True dimensions of the penis have always aroused a lot of interest in the general population, especially for penile augmentation (Azab et al., 2021; Zhang et al., 2019).

The purpose of the present study was to assess men's penile dimensions in a study in which the men would presumably be motivated to report accurate information about their penis size. Then, the aim of the present study was to evaluate the size of the penis at rest and in erection of Italian men. A secondary purpose was to explore the penile size differences between the various macro‐areas of Italy: North, Central, South and Islands. A tertiary objective was to investigate the relationship between penile dimensions and somatometric parameters in the same group.

## 2 MATERIALS AND METHODS

A total of 4,685 men from Italy and who have been visited at the Italian urology operating units were prospectively included in the study, which took place between January 2019 and January 2020 at the Careggi Hospital in Florence. We enrolled patients from the outpatient clinic during andrological consultation. Patients ≤ 15 years with erectile dysfunction, previous pelvic surgery, suspected hypogonadism, penile disease or deformity were excluded. Other exclusion criteria were applied as previously reported (Sanches et al., 2018). From each patient admitted to the study, basic information was collected: men completed demographic items (age, height, weight and height, habit of smoking, residence, and sexual orientation). Each patient was given detailed and illustrated directions on how to measure their penis (erect length and circumference) in flaccidity and in erection, from the lower base to the distal penile tip. Most men measured their penis while alone, using hand stimulation to become erect. All measurements were performed under similar environmental conditions (air‐conditioned room and at temperatures varying from 23 to 25°C). Penile length was measured along the dorsum of the penis by a ruler with millimetre markings, with the patients standing up. The penile dimensions assessed were penile length from the pubo‐penile skin vertex, depressing the pubic fat, to the extremity of the glans, with the ruler placed against the dorsal part of the penis and the circumference, the diameter at the midpoint of the penile shaft, in flaccidity and in erection (Suppl. Figure S1). All participants’ ages were recorded. Their height and weight were measured and recorded, and their BMI (ratio of weight in kilograms to height in meters squared) was calculated.

The study has been carried out in accordance with the Declaration of Helsinki for experiments involving humans and an informed consent has been signed from each patient.

### 2.1 Statistical analysis

Continuous variables are presented as median and interquartile range (IQR) and were compared by the Student's independent *t*‐test or the Mann‐Whitney *U*‐test based on their normal or not‐normal distribution, respectively (normality of variables’ distribution was tested by the Kolmogorov‐Smirnov test). Categorical variables were tested with the chi‐square test. Linear regression was used to evaluate whether the height can be associated with penile length and circumference. All statistical analyses were completed using SPSS version 17 (Statistical Package for Social Science. SPSS Inc. Released 2008. SPSS Statistics for Windows, version 17.0. (SPSS Inc., Chicago, IL). For all statistical comparisons, a significance level of *p* < .05 was considered to show differences between the groups by Wilcoxon's signed rank test.

## 3 RESULTS

**FIGURE 1:**
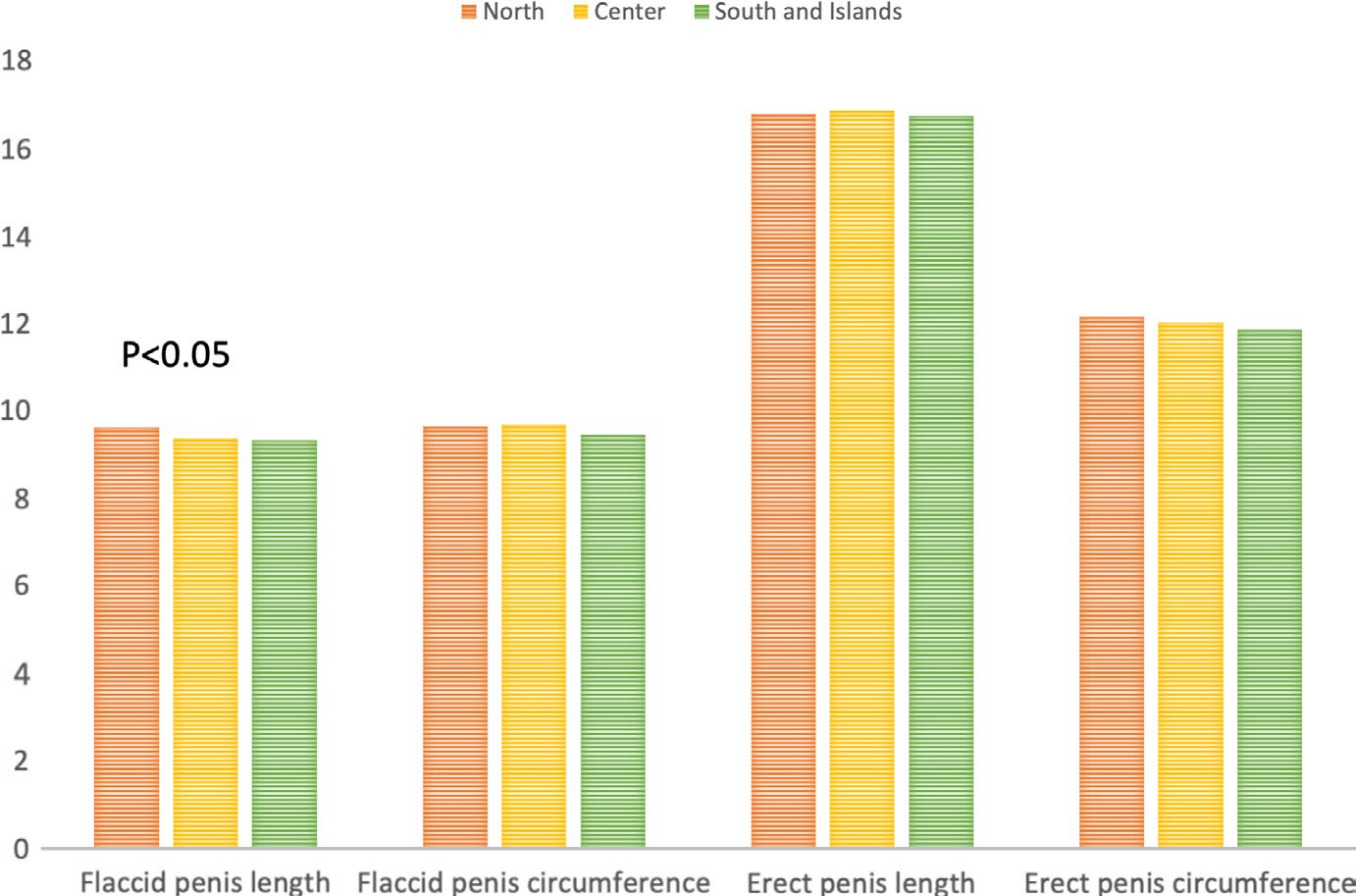
Mean penile dimension in Italy

**FIGURE 2:**
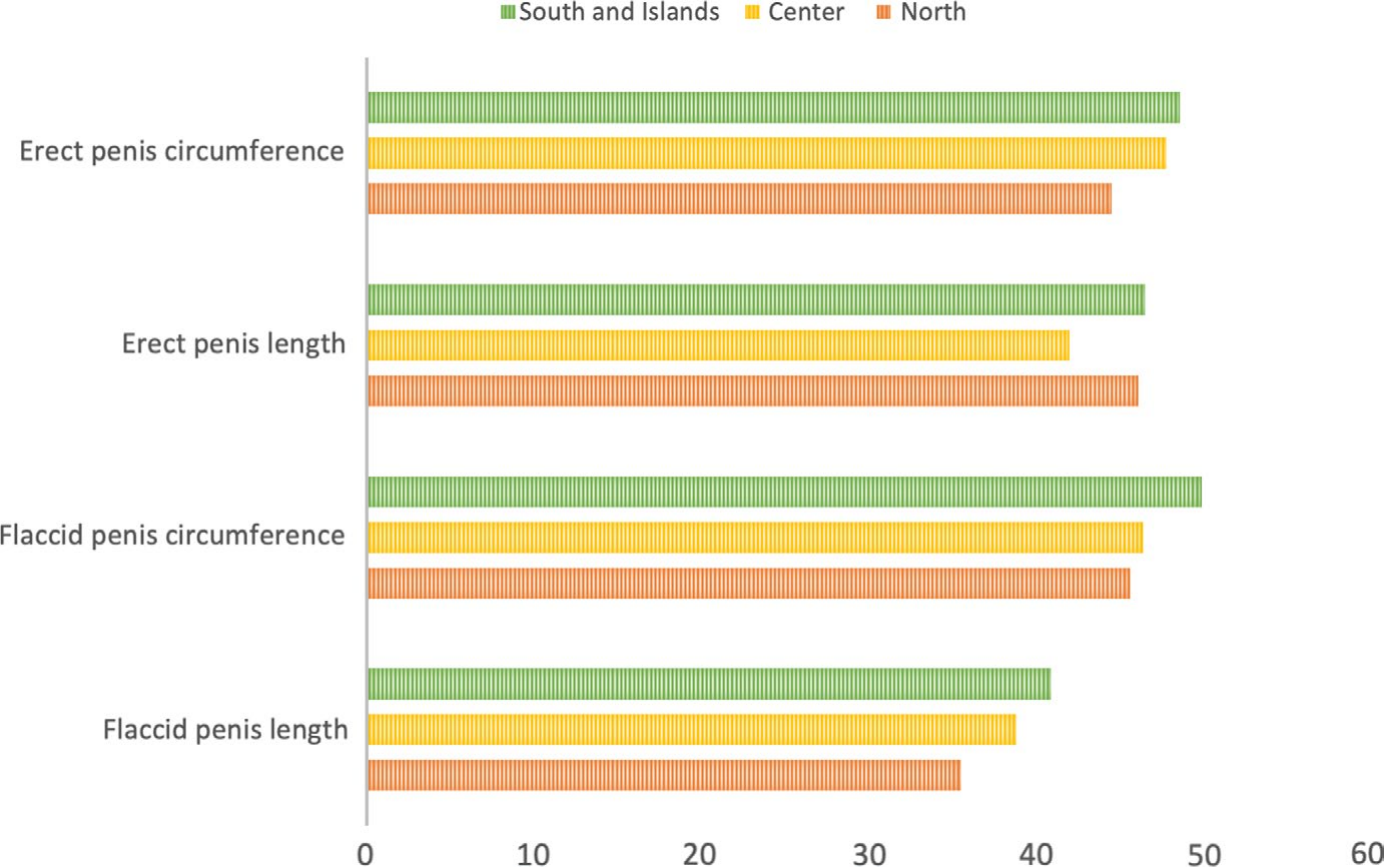
Rate of patients with penile somatometrics below the median patients by geographical area

**FIGURE 3:**
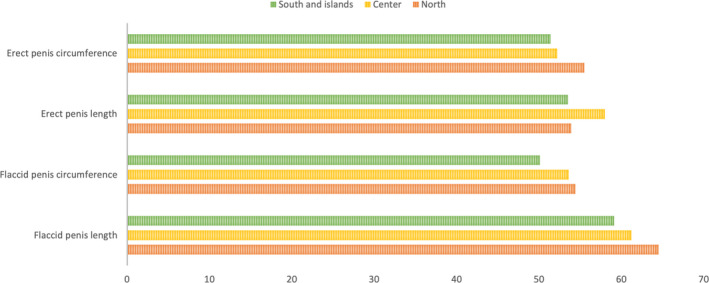
Rate of patients with penile somatometrics above the median patients by geographical area

**FIGURE 4:**
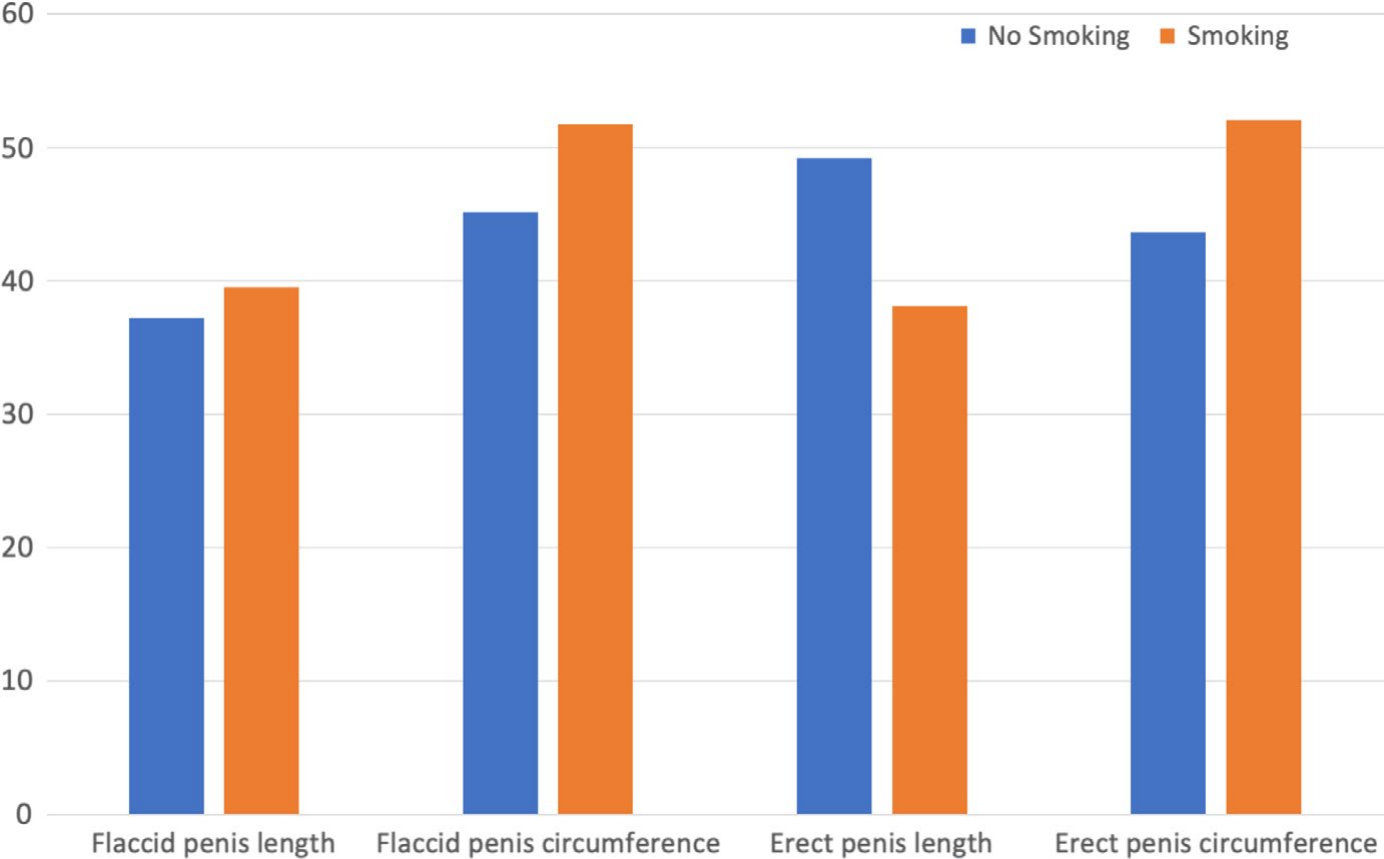
Rate of patients with penile somatometrics below the median patients by smoking habit

**FIGURE 5:**
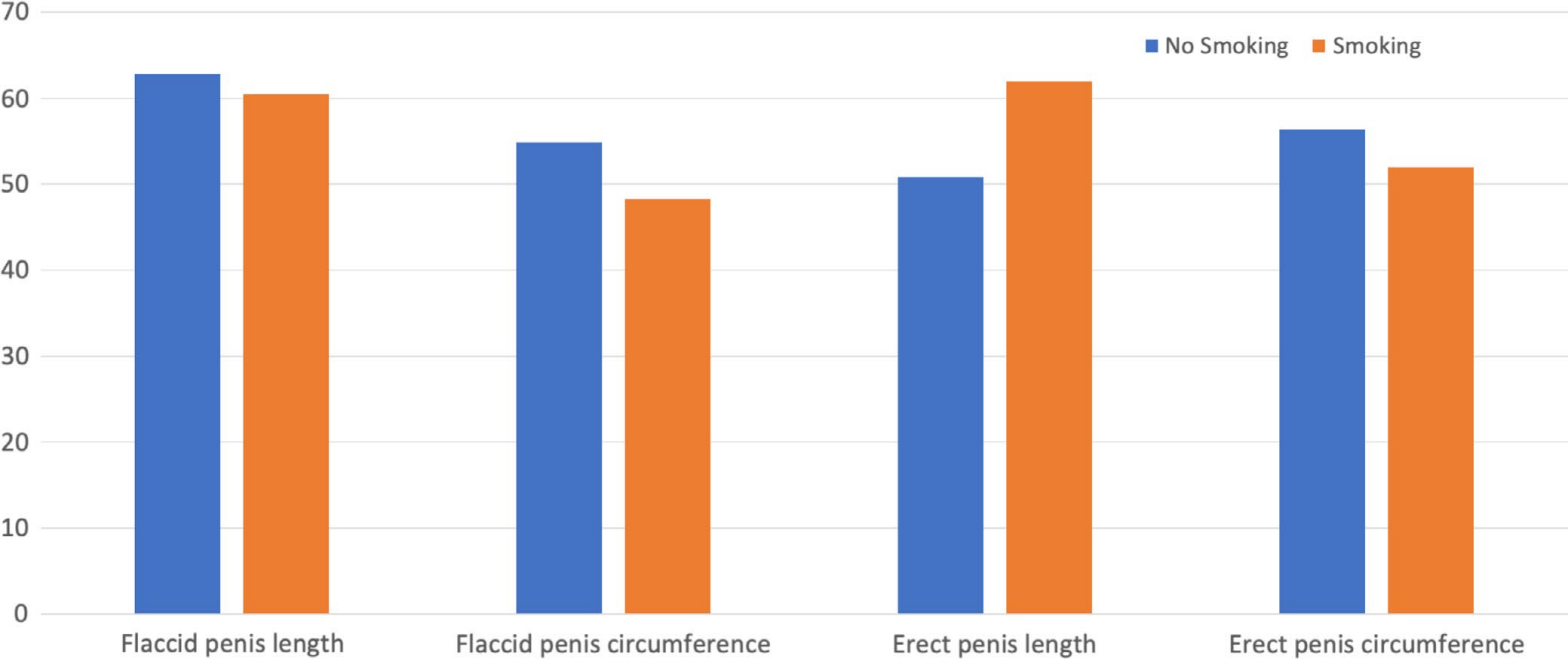
Rate of patients with penile somatometrics above the median patients by smoking

Baseline characteristics of the population are shown in Table 1. The mean (standard deviation [*SD*]) age was 19 (6.2) years, mean (*SD*) height was 177.9 (10.96) cm and mean (*SD*) weight was 72.74 (26.3) kg and mean (*SD*) BMI was 23.29 (9.68) Kg/m^2^. Smoking patients were 1,582 (33.8%) while non‐smoking patients were 3,103 (66.2%). 2,208 patients (47.1%) came from North of Italy, 907 patients (19.4%) came from Italy's centre and 1,570 patients (33.5%) came from South and Islands of Italy. We analysed sexual orientation: 11 (0.2%) patients were asexual, 4,067 (87.1%) patients were heterosexual, 165 (3.5%) were homosexual, bisexual patients were 416 (8.9%) and pansexual patients were 11 (0.2%). Mean (*SD*) flaccid penis length was 9.47 (2.69), mean (*SD*) flaccid penis circumference was 9.59 (3.08), mean (*SD*) erect penis length was 16.78 (2.55) and mean (*SD*) erect penis circumference was 12.03 (3.82). Tables 2, 3, 4 show baseline characteristics of North, Centre, South and Islands population. The mean of penis size stratified by geographic area did not reveal statistically significant differences, except for the length of the flaccid penis (*p* <.01) (Figure 1). The percentage distributions by geographical area are shown in the Supplementary Tables S1–S4 and divided into patients above and below the median with reference to penis size (Figures 2, 3). From our data, we found that 48.2% of men in the North have a flaccid penis length above the national average, compared to 19.7% of men in the Centre and 32.1% of men in Southern Italy and in the islands (*p* < .01). Instead, for the other penile dimensions, we did not find statistical significance based on the geographical area. In addition, we have developed contingency tables for the analysis of patients with a smoking habit and a median of the penis size. Our results indicate that 60.5% of smoking patients have a median flaccid penis length above median compared to 62.8% of non‐smoking patients. However, these data are not statistically significant (*p*‐value .08). Only 48.3% of smoking patients have a median flaccid penis circumference above median compared to 54.9% of non‐smoking patients (*p* value < .05). Furthermore, 61.9% of smoking patients have a median erect penis circumference above median compared to 50.8% of non‐smoking patients (*p* < .01). Also, 48.0% of smoking patients have a median erect penis circumference above median compared to 56.4% of non‐smoking patients (*p* < .01) (Supplementary Tables S5–S8 and Figures 4 and 5).

**TABLE 1:** Epidemiological data of the cohort

**Patients, *N* = 4,685**	
Age (years), mean (*SD*)	19 (6.2)
Height (cm), mean (*SD*)	177.99 (10.96)
Weight (Kg), mean (*SD*)	72.74 (26.3)
BMI (kg/m^2^) mean (*SD*)	23.29 (9.68)
**Smoking, *n* (%)**	
No	3,103 (66.2)
Yes	1582 (33.8)
**Area of origin, *n* (%)**	
North	2,208 (47.1)
Centre	907 (19.4)
South and Island	1,570 (33.5)
**Sexual Orientation, *n* (%)**	
Asexual	11 (0.2)
Heterosexual	4,067 (87.1)
Homosexual	165 (3.5)
Bisexual	416 (8.9)
Pansexual	11 (0.2)

Abbreviation: BMI, Body mass index.

**TABLE 2:** Baseline characteristics of the north Italian population in the study

**Patients, *N* = 2,208**	
Age (years), mean (*SD*)	20.47 (6.16)
Height (cm), mean (*SD*)	178.60 (8.43)
Weight (Kg), mean (*SD*)	72.74 (13.28)
BMI (kg/m^2^) mean (*SD*)	22.90 (4.72)
**Smoking, *n* (%)**	
No	1522 (68.9)
Yes	686 (31.1)
**Sexual Orientation, *n* (%)**	
Asexual	3 (0.1)
Heterosexual	1914 (86.9)
Homosexual	72 (3.3)
Bisexual	207 (9.4)
Pansexual	6 (0.3)
**Penile Dimensions, cm median (IQR)**	
Flaccid penis length	10 (8–11)
Flaccid penis circumference	10 (8–11)
Erect penis length	17 (15–18)
Erect penis circumference	13 (10–15)

**TABLE 3:** Baseline characteristics of the centre Italian population in the study

**Patients, *N* = 907**	
Age (years), mean (*SD*)	19.83 (5.11)
Height (cm), mean (*SD*)	178.38 (7.36)
Weight (Kg), mean (*SD*)	73.41 (13.13)
BMI (kg/m^2^), mean (*SD*)	23.04 (3.78)
**Smoking, *n* (%)**	
No	589 (64.9)
Yes	318 (35.1)
**Sexual Orientation, *n* (%)**	
Asexual	2 (0.2)
Heterosexual	802 (88.7)
Homosexual	31 (3.4)
Bisexual	67 (7.4)
Pansexual	2 (0.2)
**Penile Dimensions, cm median (IQR)**	
Flaccid penis length	9 (8–11)
Flaccid penis circumference	10 (7–12)
Erect penis length	17 (16–18)
Erect penis circumference	13 (9–14)

**TABLE 4:** Baseline characteristics of the South and Islands Italian population in the study

**Patients, *N* = 1,570**	
Age (years), mean (*SD*)	20.64 (6.71)
Height (cm), mean (*SD*)	176.74 (15.13)
Weight (Kg), mean (*SD*)	73.68 (27.47)
BMI (kg/m^2^), mean (*SD*)	24.02 (15.63)
**Smoking, *n* (%)**	
No	992 (63.2)
Yes	578 (36.8)
**Sexual Orientation, *n* (%)**	
Asexual	6 (0.4)
Heterosexual	1,351 (86.4)
Homosexual	62 (4.0)
Bisexual	142 (9.1)
Pansexual	3 (0.2)
**Penile Dimensions, cm median (IQR)**	
Flaccid penis length	9 (7–11)
Flaccid penis circumference	10 (7–11)
Erect penis length	17 (15–18)
Erect penis circumference	13 (9–15)

At the linear regression analysis, height was associated with flaccid penis length (*β* = 0.04; *p*‐value = .01), erect penis length was (*β* = 0.05; *p*‐value < .01) and erect penis circumference was (*β* = 0.06; *p*‐value < .01).

We also demonstrated an association between BMI and flaccid penis circumference (*β* = 0.08; *p*‐value < .01), and erect penis length was (*β* = −0.07; *p*‐value < .01). Finally, weight was associated with flaccid penis length (*β* = −0.06; *p*‐value < .01).

## 4 DISCUSSION

To the best of our knowledge, no recent study has comprehensively assessed penile size in Italian men. The most recent study conducted in Italy dates back to 2001 out of 3,300 patients by Ponchietti et al. and reports media (*SD*) flaccid length 9 (2), media flaccid circumference 10 (0.75), but the size of the erect penis has not been investigated (Ponchietti et al., 2001). Many studies have investigated the size of the penis in the past years in the world, but many of these have the bias of being very heterogeneous and with a very small sample. One of the largest is that of Herbenick et.al that evaluated the erect penis size of 1661 sexually active men in the USA. It showed that mean erect penis length was 14.15 cm (*SD* = 2.66; range = 4 to 26 cm) and mean circumference of the erect penis was 12.23 cm (Herbenick et al., 2014). Another great study is that of Söylemez, a study conducted on 2,276 young Turkish men. In Söylemez's study, the mean flaccid, fully stretched and circumferential length of the participants penises were 8.95 ± 1.04, 13.98 ± 1.58 and 8.89 ± 0.86 cm, respectively (Söylemez et al., 2012), but the size of the erect penis has not been investigated in this either. Establishing what the normal size of the penis is very important to have a yardstick for men, who often tend to underestimate their size.

A recent article from health records of 14,597 Vietnamese men found median values are 9.03 cm for flaccid length, 14.67 cm for stretched length, 8.39 cm for mid‐shaft circumference and 2.86 cm for unaroused glans diameter (Nguyen Hoai et al., 2021) and specifically, men with erectile dysfunction had a greater value in all penile dimensions compared with other groups (health screening group and other disease groups).

Putting together our findings with other reports, our data were similar with those from Western Asians (8.96 ± 1.13 cm) (Aslan et al., 2011) (Sengezer et al., 2002) and USA (9.01 ± 2.15 cm) (Wessells et al., 1996).

It is important to underline that associations between penile size and somatometric parameters papers still remain controversial. Results from a systematic review with up to 15,521 males in 20 studies showed that all somatometric correlations were either inconsistent or weak while the most reliable was the association flaccid stretched length and height (Veale et al., 2015).

Although all these premises, body acceptance and self‐satisfaction are important in confidence and could play a role on sexual life (Veale, Miles, Read, Troglia, Wylie, et al., 2015)(Veale et al., 2014).

Furthermore more, consultation for Peyronie's disease is extremely important in order to give expectation for penile length after surgery, since it represents one of the most important outcome (Russo et al., 2019)(Falcone et al., 2020)(Cocci et al., 2020)(Cocci et al., 2018). For all these reasons, updated results on penile dimensions remain crucial for clinical and psychological assistance of patients with sexual dysfunctions.

Patients with impression of small penis may feel anxious, less capable of maintaining erections, resulting in an impact on sexual frequency and ejaculations. Knowing the real average size of the penis is of growing interest to perform a correct diagnostic evaluation and therapeutic choice in patients with concerns about its penis adequacy.

The study of Veale et al proposed a nomogram useful in clinical and therapeutic settings to counsel men and for academic research. Moreover, it is important to underlie different limitations for penile measurement.

In particular, temperature, level of arousal and previous ejaculation could also affect the penile dimensions. Using a disposable tape measure, a participant should have three parameters measured in the flaccid state: circumference (girth) of the penile, mid‐shaft; length from suprapubic skin to distal glans (skin‐to‐tip); and pubis to distal glans (bone‐to‐tip) (Veale, Miles, Bramley, et al., 2015).

Before concluding, we should address some limitations. Firstly, measurements have not been conducted by the physician but this would have been unethical in an outpatient setting. In fact, performing the measurement during the visit would need the use of drug for the induction of erection or even self‐made masturbation or during anaesthesia (Akyüz, 2020). Secondly, patients only measured the penis one time with possible error of measurement. Thirdly, we did not perform a comparison with other countries. Finally, we did not evaluate the impact of smoking duration and quantity with penile size.

## 5 CONCLUSION

Our study, therefore, showed that there are no statistically significant differences for penis size in Italy in the North, Central, South and Islands macro‐areas, except for the length of the flaccid penis, which was greater in the North and lower in Central Italy. Our data showed that smoking patients are more likely to have a flaccid and erect penis circumference below average. In addition, we have shown that somatometrics characteristics matter. In particular, the height is proportional to the length of the penis in flaccidity and in erection, and to the circumference in erection. Furthermore, the increase in BMI leads to a reduction in the length of the erect penis, as well as weight gain reduces the length of the flaccid penis.

## CONFLICT OF INTEREST

None.

## AUTHOR CONTRIBUTIONS

Conceptualization: A.C. Data curation: A.C.; D.G.; C.T: Formal analysis: M.D.; G.I.R. Methodology: A.C.; D.G:. Writing original draft: M.D.; G.I.R.; G.C.; G.C.; G.M.; G.P.; F.D. Writing review & editing: A.C.; M.D.; G.I.R.

## ETHICAL APPROVAL

All the study procedures were carried out in accordance with the Declaration of Helsinki (2013) of the World Medical Association and participants provided written informed consent after accepting to participate.

## Supporting information

Fig S1

Table S1‐S8

## Data Availability

The data that support the findings of this study are available from the corresponding author upon reasonable request.
