# Supplementary figures and images for: Penile length and circumference dimensions: A large study in young Italian men
Source: Andrologia. 2021 Mar 21;53(6):e14053. doi: 10.1111/and.14053 (PMC8243978; doi:10.1111/and.14053)

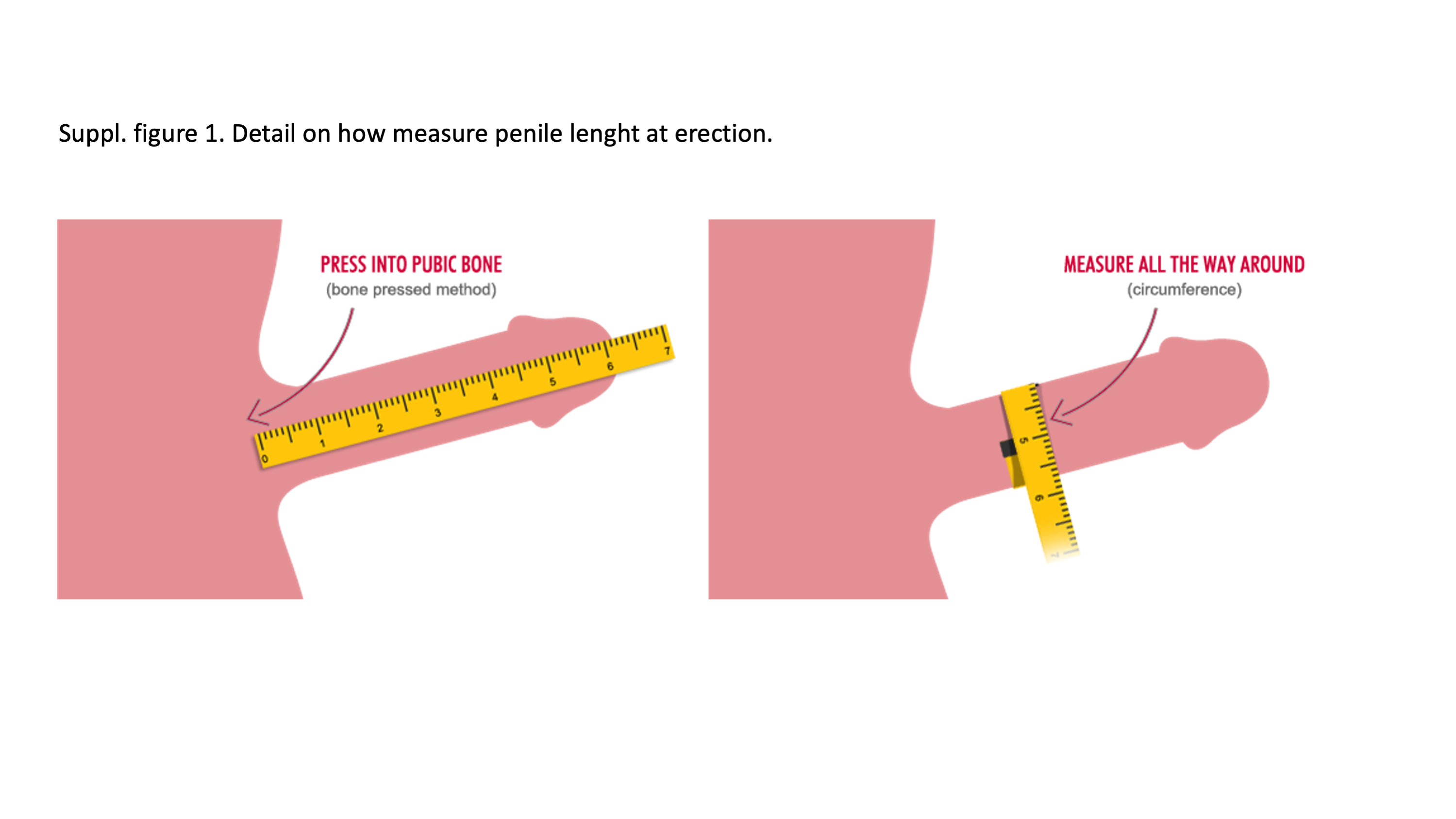

Supplement: Supplementary file 1 — Fig S1 [file AND-53-e14053-s002.tiff]
