# Supplementary material for: Penile length and circumference dimensions: A large study in young Italian men
Source: Andrologia. 2021 Mar 21;53(6):e14053. doi: 10.1111/and.14053 (PMC8243978; doi:10.1111/and.14053)
Supplement: Supplementary file 2 — Table S1‐S8 [file AND-53-e14053-s001.docx]

**Suppl. Table S1.** Contingency table for the analysis of median penis length in flaccidity by geographical area

|  | **< 9 (Median)** | **> 9 (Median)** | **pvalue** |
| --- | --- | --- | --- |
| **North** | 692 (43.4) | 1255 (48.2) | **0.006** |
| **Center** | 325 (20.4) | 512 (19.7) |  |
| **South e Islands** | 579 (36.3) | 837 (32.1) |  |

**Suppl. Table S2.** Contingency table for the analysis of median penis circumference in flaccidity by geographical area

|  | **< 10 (Median)** | **> 10 (Median)** | **pvalue** |
| --- | --- | --- | --- |
| **North** | 697 (45.0) | 830 (47.9) | 0.09 |
| **Center** | 300 (19.4) | 347 (20.0) |  |
| **South e Islands** | 551 (35.6) | 554 (32.0) |  |

**Suppl. Table S3.** Contingency table for the analysis of penis length in erection by geographical area

|  | **< 17 (Median)** | **> 17 (Median)** | **pvalue** |
| --- | --- | --- | --- |
| **North** | 859 (47.1) | 1005 (45.8) | 0.1 |
| **Center** | 329 (18.0) | 455 (20.8) |  |
| **South e Islands** | 635 (34.8) | 732 (33.4) |  |

**Suppl. Table S4.** Contingency table for the analysis of median penis circumference in erection by geographical area

|  | **< 13 (Median)** | **> 13 (Median)** | **pvalue** |
| --- | --- | --- | --- |
| **North** | 771 (44.8) | 963 (48.7) | 0.06 |
| **Center** | 340 (19.8) | 371 (18.7) |  |
| **South e Islands** | 610 (35.4) | 645 (32.6) |  |

**Suppl. Table S5.** Rates of smoking status according to median penis length in flaccidity.

|  | **< 9 (Median)** | **≥ 9 (Median)** | **pvalue** |
| --- | --- | --- | --- |
| **Smoking** |  |  | 0.08 |
| **No** | 1026 (37.2) | 1730 (62.8) |  |
| **Yes** | 570 (39.5) | 874 (60.5) |  |

**Suppl. Table S6.** Rates of smoking status according to median circumference length in flaccidity

|  | **< 10 (Median)** | **≥ 10 (Median)** | **pvalue** |
| --- | --- | --- | --- |
| **Smoking** |  |  | **0.00** |
| **No** | 1003 (45.1) | 1222 (54.9) |  |
| **Yes** | 545 (51.7) | 509 (48.3) |  |

**Suppl. Table S7.** Rates of smoking status according to median penis length in erection.

|  | **< 17 (Median)** | **≥ 17 (Median)** | **pvalue** |
| --- | --- | --- | --- |
| **Smoking** |  |  | **0.00** |
| **No** | 1301 (49.2) | 1345 (50.8) |  |
| **Yes** | 522 (38.1) | 847 (61.9) |  |

**Suppl. Table S8.** Rates of smoking status according to median penis circumference in flaccidity

|  | **< 13 (Median)** | **≤ 13 (Median)** | **pvalue** |
| --- | --- | --- | --- |
| **Smoking** |  |  | **0.00** |
| **No** | 1060 (43.6) | 1370 (56.4) |  |
| **Yes** | 661 (52.0) | 609 (48.0) |  |
